# Time Series UAV Image-Based Point Clouds for Landslide Progression Evaluation Applications

**DOI:** 10.3390/s17102378

**Published:** 2017-10-18

**Authors:** Abdulla Al-Rawabdeh, Adel Moussa, Marzieh Foroutan, Naser El-Sheimy, Ayman Habib

**Affiliations:** 1 Department of Earth and Environmental Science, Yarmouk University, Irbid 21163, Jordan; 2 Department of Geomatics Engineering, University of Calgary, Calgary, AB T2N 1N4, Canada; amelsaye@ucalgary.ca (A.M.); elsheimy@ucalgary.ca (N.E.-S.); 3 Department of Electrical Engineering, Port-Said University, Port Said 42523, Egypt; 4 Department of Geography, University of Calgary, Calgary, AB T2N 1N4, Canada; foroutam@ucalgary.ca; 5 Lyles School of Civil Engineering, Purdue University, West Lafayette, IN 47907, USA; ahabib@purdue.edu

**Keywords:** change detection, landslide dynamics, 3D dense matching, unmanned aerial vehicle (UAV), image-based registration

## Abstract

Landslides are major and constantly changing threats to urban landscapes and infrastructure. It is essential to detect and capture landslide changes regularly. Traditional methods for monitoring landslides are time-consuming, costly, dangerous, and the quality and quantity of the data is sometimes unable to meet the necessary requirements of geotechnical projects. This motivates the development of more automatic and efficient remote sensing approaches for landslide progression evaluation. Automatic change detection involving low-altitude unmanned aerial vehicle image-based point clouds, although proven, is relatively unexplored, and little research has been done in terms of accounting for volumetric changes. In this study, a methodology for automatically deriving change displacement rates, in a horizontal direction based on comparisons between extracted landslide scarps from multiple time periods, has been developed. Compared with the iterative closest projected point (ICPP) registration method, the developed method takes full advantage of automated geometric measuring, leading to fast processing. The proposed approach easily processes a large number of images from different epochs and enables the creation of registered image-based point clouds without the use of extensive ground control point information or further processing such as interpretation and image correlation. The produced results are promising for use in the field of landslide research.

## 1. Introduction

Changes and displacements are fundamental indications of the Earth’s surface’s mass movements, such as landslides, soil creep, and rock slides, which are caused by either human activity or natural processes. Detecting surface changes is of great importance for the reduction of natural hazards and dynamic monitoring in environmental management. Automatic detection of the Earth’s surface change is a useful tool for providing efficiency regarding time and money, providing safe data collection, and providing reliable information for decision making. This is important for a variety of applications ranging from large scale investigations, such as land-use change analysis, disaster monitoring, and environmental modelling [1,2,3], to small scale investigations such as small bedform movements and the deformation of small objects [4,5]. Earth movement can be classified into several typologies, such as landslides which are the focus of this study. A landslide is a combination of layers having contrasting and gradational physical properties [6]. Other natural disasters often trigger landslides, such as earthquakes, a temporal concentration of heavy rainfall, or volcanic eruptions. Landslides are recognized as a natural disaster of significance that occurs extensively in almost every country of the world [7,8]. The monitoring of landslides requires on-going assessments of the extent and the rate of horizontal (horizontal scarp progression or scarp retreated) and vertical (depth) displacements of the surface’s terrain. The timely and accurate detection and quick identification of landslides is crucial for adopting appropriate mitigation measures and efficient decision-making strategies especially when the landslide areas are less than 0.02 km^2^ [9,10].Identifying the smallest detectable landslide scarp is important because small landslide areas are very likely to expand under heavy rainfall conditions.

Traditional field survey methods (e.g., using total station, GPS, and geological compass) for measuring the factors that lead to landslides, do not meet the required cost, time efficiency, and safety parameters. Landslide monitoring of all classes [11] (e.g., rockslide/rockfall and creep landslide) requires continual assessment of the extent, rate of displacement, surface topography, and detection of fissure structures potentially related to fracture processes [12]. Previous landslide mapping methods for detecting landslide scarps are typically based on ground surveys with a global positioning system (GPS) and can be deployed with a total station. These types of measurements provide sparse spatial coverage, resulting in the omission of fine-scale terrain structures in the digital elevation models (DEMs) [13]. Image analysis using object-to-background separation or a simple subtraction technique between the images for change detection is plagued by misinformation that is caused by shadows or other local illumination problems [14]. Recent developments of both passive and active remote sensing tools and techniques, such as unmanned aerial vehicles (UAVs), airborne light detection and ranging (LiDAR) and terrestrial laser scanning (TLS) technology provide safe, timely and accurate information, and have become increasingly popular in different geoscience disciplines [15,16,17].

A key step in the processing chain is the ability to distinguish changed surfaces from unchanged surfaces by using two or more image sets acquired at different epochs. Currently, generated dense 3D point clouds require additional processing steps to arrive at a change detection analysis, including the registration process, where all point clouds are transformed to the same coordinate system. The registration approaches found within literature can be categorized into (1) target-based methods, [18]; (2) feature-based methods [19]; (3) direct geo-referencing methods that are based on integrated Global Navigation Satellite System/inertial navigation system (GNSS/INS) [20]; and (4) surface/point cloud matching techniques using all available point clouds (i.e., the iterative closest point (ICP) method and its variants) [21].

In target-based methods, objects or targets are placed within the scanner’s field of view and are not moved throughout any of the scanning positions. This method sometimes requires cumbersome equipment, as well as time-consuming target set up. Furthermore, these targets are not always visible in successive scans and require replacements for registration. In addition, the initial alignment (coarse alignment) between the involved point cloud models is manually achieved, and the identification of a specific point in the resulted point cloud models, even from the TLS or an image-based point cloud, is difficult and unreliable as the surface model’s footprints are irregularly-distributed [22]. A drawback of the TLS system is that it is not exactly known which point on an object causes the reflection, and it is therefore difficult to exactly identify a point in the point cloud as a tie point. A fine registration is required through point-based registration algorithms. The feature-based method is effective for registering the point clouds of industrial facilities where many objects with a regular geometric shape exist [18]. Direct geo-referencing (DG) based on GPS/INS systems are applicable for airborne laser scanners [23], mobile terrestrial laser scanners [24], and static terrestrial laser scanners [25]. DG reduces or eliminates the requirement for ground control points (GCPs), which is beneficial for inaccessible areas and reduces the mapping cost [26]. However, the incorporation of direct geo-referencing sensors imposes additional costs on the scanning system, and the quality of the alignment is also dependent on the accuracy of the utilized GNSS/INS units. In the case of less accurate GNSS/INS-based position and orientation information, a point-based registration is applied as a further step to achieve the fine alignment between the point clouds. Additionally, in remote and narrow areas, such as mountainous areas where landslides are likely to occur, the availability of a stable GPS signal and multipath could be a further constraint. A surface/point cloud matching technique, the iterative closest point (ICP) method, is commonly used for the registration of a point cloud and has many variations. It requires overlapping areas with diverse geometry between data sets and a reasonable initial estimation of transformation parameters. The iterative closest projected point algorithm (ICPP) is another variant of the well-known ICP method [27], which aimed to minimize the distance between a point in one scan and its projection on the plane defined by the closest three points in another scan. Without quality initial parameters and large overlapping data sets, the ICP method, and its variants, can fail to estimate reliable registration parameters and its slow algorithm requires the use of all available points in the datasets. Moreover, it relies on an interactive approach, requiring users to guide the initial coarse alignment process by manually providing correspondences before running the final fine registration [28].

Immerzeel et al., deployed a UAV over a debris-covered Himalayan glacier in Nepal [29]. Based on stereo imaging from two campaigns in 2013, they derived highly-detailed ortho-mosaics and DEMs by using the structure from motion (SfM) algorithm. Differential GPS observations are collected in the field in order to geometrically correct the orthophoto. Founded on DEM differencing and manual feature tracking, they derived the mass loss and the surface velocity of the glacier with a high spatial accuracy. Wang et al., reconstructed the 3D surface of a detritus area located at the Zijin Mine in the Fujian Province, China [30]. They implemented different algorithms, such as the SfM system and the patch-based multiview stereo (PMVS) system, to generate a dense 3D point cloud from the UAV images. They used 17 GCPs to geo-reference a 3D reconstruction point cloud, and the accuracy of the 3D geometry was evaluated by using both the GCPs and the TLS point cloud. The UAV point cloud accuracy was first evaluated at a point level by comparing the absolute coordinates between the UAV point cloud and the GCPs. Lucieer et al., used a UAV platform equipped with a standard digital camera and GPS to collect multitemporal sets of extremely high-resolution RGB images over the active Home Hill landslide in Tasmania [15]. Multi-view stereopsis (MVS) and SfM methods were used to convert the overlapping images into 3D point clouds, DEMs, and ortho-mosaics. The horizontal landslide displacements were detected by using a semi-automatic image correlation technique (Co-registration of Optically Sensed Images and Correlation algorithm COSI–Corr) after converting the two DEMs from different epochs into shaded relief images. The algorithm successfully quantified the movements of the large pieces of ground material, but was less successful when mapping the main landslide scarp. Wujanz analyzed the significance/informative value of quality measures in surface-based registration processes by using dataset effects of deformation onto commercial software (GFaI Final Surface 3.0.5, GFaI Gesellschaft zur Förderung angewandter Informatik e. V, Berlin, Germany; Leica Cyclone 7.1, Leica Geosystems AG, Heerbrugg, Switzerland; and Geomagic Studio 12, Raindrop Geomagic, Inc., Durham, North Carolina, USA) and scientific (four-points congruent sets algorithm) applications [31]. The results indicated that none of the implemented quality measures led to a definitive conclusion that the “best” result had been achieved.

The present study explains the mission planning and data acquisition process, followed by a discussion of how UAV image-based point cloud data can be used in the change detection of small magnitudes (up to few cm) through a direct point cloud comparison. An analysis of the alignment of multiple generated point clouds into a common coordinate system using the proposed registration method is provided as well as an evaluation of the qualitative and quantitative aspects of the proposed registration method versus the ICPP. Finally, the application of 3D point correspondence using a point-to-patch comparison for detecting volumetric surface changes is presented as well as the change displacement rate in the horizontal direction based on comparisons extracted from landslide scarps from image-based point clouds generated from multiple epochs.

## 2. Study Area

**Figure 1:**
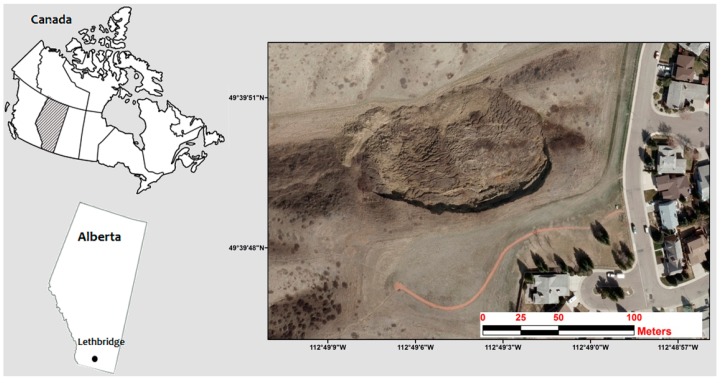
Geographic location of study area (southern part of Lethbridge, Alberta Canada).

The study site is located in a highly dynamic site that is affected by soil creep landslides within a rural area, lying on the southern edge of the City of Lethbridge, Alberta, Canada. This area is situated between 112°49′0.35″ W and 112°49′8.41″ W Longitude and 49°39′53.83″ N and 49°39′46.08″ N Latitude. It extends approximately 200 m east to west and is roughly 110 m at its widest point north-to-south. It maintains elevations of between 924 m at the south end and 872 m at the north end, with slope angles of more than 40° (Figure 1).

This area has a semi-arid climate with an average maximum temperature of 12.3 °C and an average minimum temperature of −1.1 °C. It has an average of 264 dry days annually and an annual precipitation of between 365 mm and 386.3 mm, which makes Lethbridge the 11th driest city in Canada [32]. The mean relative humidity hovers between 69% and 78% during the mornings throughout the year, but the mean relative humidity in the afternoon is more uneven, ranging between 38% in August to 58% in January [33]. The land-cover types in the region include landslide areas, roads, bare soil, and residential districts. Non-cultivated parts of the area are covered by the typical prairie flora.

The landslide activity within the study area occurs along the coulees draining into the Oldman River. These slides are relatively large in terms of the area and they typically involve a combined rotational/translational type of failure movement. This movement generally follows a pattern, occurring along one or more of the curved failure surfaces with a downward motion near the top and an outward motion towards the bottom. This feature likely moves more when triggered by one of, or a combination of, the following circumstances: a rise in the local groundwater table; a fill placement along the crest of the coulees; an uncontrolled discharge of surface water runoff during the heavy spring rains; or a significant snowmelt runoff.

## 3. Methodology

**Figure 2:**
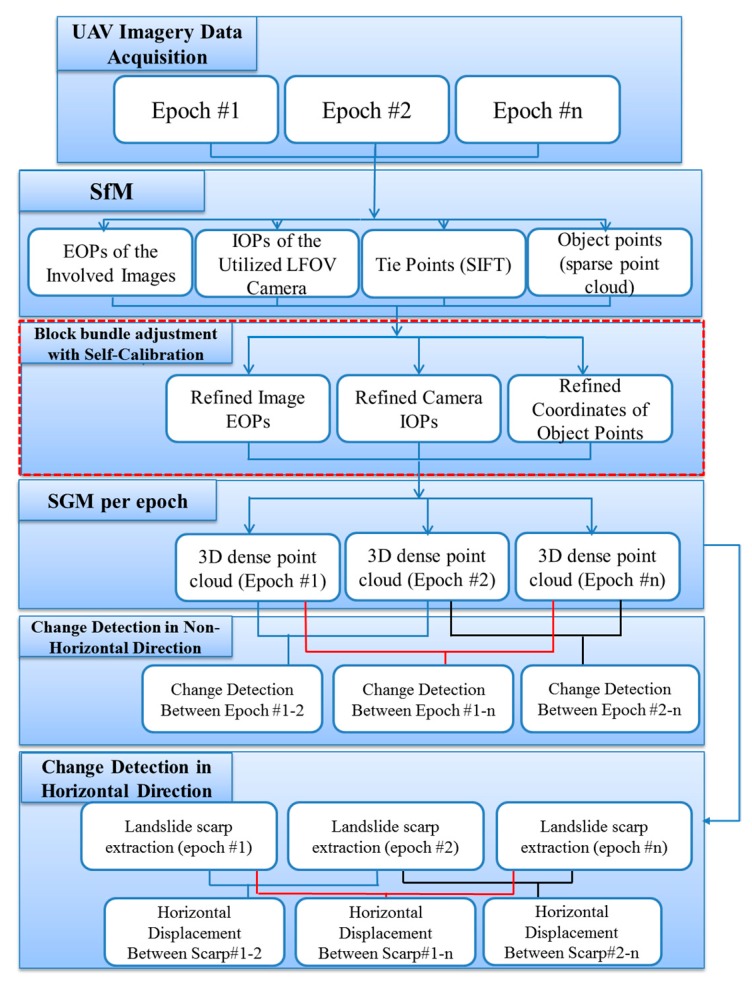
Overview of the workflow from the acquired unmanned aerial vehicle (UAV) image-based datasets to the change detection analysis.

The procedural workflow for detecting changes between two UAV image-based point clouds collected at different epochs is shown in Figure 2. The methodology used to detect changes between two or more UAV image-based point clouds consists of six steps. The first step includes the customization of the UAV equipment by adding a GPS logger and a low-cost large-field-of-view (LFOV) digital action camera to facilitate the capture of high-resolution geo-tagged images. Due to the coarse accuracy of the on-board GPS receiver (e.g., ±5–±10 m), only the geo-tagged positions of the images are used as initial values for the procedures that follow. The second step is a SfM procedure where the preliminary image exterior orientation parameters (EOPs), the camera interior orientation parameters (IOPs), and the ground coordinates of the tie points are estimated using the images from all the observed epochs. Note that the conjugate points are collected and matched via the scale-invariant feature transform (SIFT) detector and descriptor. In the third step, the parameters estimated in the SfM procedure are refined through a block bundle adjustment with self-calibration. The fourth step is a semi-global dense matching procedure to generate a dense 3D point cloud for each observed epoch using the images captured only at that particular epoch. As a result of the bundle adjustment procedure, the separate point clouds are effectively co-registered to a common reference frame. The fifth step includes the computation of the normal distances between any two consecutive point clouds and is examined to evaluate the proposed registration procedure by comparing the non-active patches within the monitored area of interest. Since these non-active sub-areas are stationary, the computed normal distances theoretically should be close to zero. A 3D point correspondence is applied using a point-to-patch comparison for detecting volumetric surface changes. Finally, the change displacement rate in the horizontal direction is explained based on the comparisons extracted from the landslide scarps from point clouds created over multiple epochs.

### 3.1. Mission Planning and Data Collection

**Figure 3:**
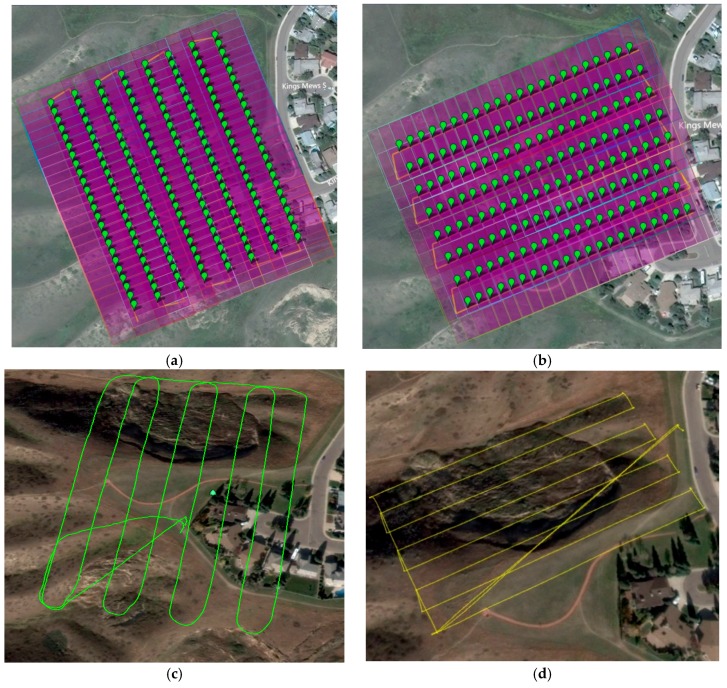
Graphical interface of the image capturing mission flight plan designed to cover study area, Lethbridge, Alberta. (**a**) North–south programmed flight path; (**b**) east–west programmed flight path; (**c**) north–south actual flight trajectory; (**d**) east–west actual flight trajectory.

The first step of the proposed workflow is the acquisition of the time-series UAV image-based datasets. The DJI Phantom II quad-copter used in this research was equipped with an LFOV action camera which has a lens with a 3 mm nominal focal length. Image acquisition was performed during two flights in different directions (Figure 3) for each field operation over the study area. With the camera operating at medium field-of-view mode, the flight lines were captured at a rate of five frames per second with an altitude of roughly 30 m and at a speed of 5 m/s, resulting in a ground sample distance (GSD) of roughly 1.7 cm. The GoPro camera was calibrated and tested for the stability of its internal characteristics using an indoor camera calibration test field and refined through an in-situ camera calibration. The United States Geological Society (USGS) simultaneous multi-frame analytical calibration (SMAC) distortion model was employed within the calibration procedure [34]. The camera calibration parameters consisted of the focal length (c), principal point offset (xp, yp), radial (K1, K2, and K3), and de-centric (P1 and P2) lens distortions.

All the flights were performed in automatic mode to maintain level flights, control altitude, and autonomously fly the UAV through a series of predefined waypoints, which was set to the maximum allowed (16 waypoints). The log system data were recorded at 1 Hz, which included the platform position as measured with the on-board consumer grade GPS. Due to wind drifts and other factors affecting the autopilot sensor on-board the UAV, deviations were present between the predefined and actual linear paths of the flight trajectories as depicted in Figure 3c,d. The entire data set was collected in less than half an hour.

### 3.2. A Novel Automatic UAV Image-Based Registration

The paper introduces a robust procedure for aligning temporal UAV image-based point clouds with respect to a common reference frame. The primary contribution of this method is the adoption of the general bundle block adjustment for image registration to minimize the geometric misalignment of both epochs. The proposed registration procedure is performed using the SfM approach developed by He and Habib [35]. This approach automates the process of image EOP recovery and sparse point cloud generation with respect to the mapping reference frame and is based on the following four-step process:

1. In the first step, the relative orientation parameters (ROPs) related to the stereo images from both epochs are derived using the coplanarity equations, where the closed-form Nistér five-point approach is used for the approximate values [36]. In this study, these conjugate point features are automatically identified through a SIFT detector and descriptor [37]. The ROP are estimated by choosing an arbitrary local coordinate system (i.e., arbitrarily fixing the position and orientation of one of the images in the block and the base distance to a neighboring image station). Thus, the ROPs are established using an arbitrary defined datum where the origin and orientation are established by the fixed image and the scale is established through the set base.
2. Once the ROPs of all possible stereo-pairs are estimated, an incremental approach developed by He and Habib [35] is used for the initial recovery of the images EOPs from both epochs. This incremental approach is initiated by defining a local coordinate frame. Then, all the images are sequentially augmented into a final image block or trajectory.
3. As the derived sparse point cloud from both epochs using the SfM approach is only defined in an arbitrary local coordinate system, an absolute orientation process must be applied for transforming the derived sparse point cloud as well as the estimated image EOPs relative to the mapping reference frame. In this study, we use GPS measurements which are recorded at each image exposure time by a consumer-grade GPS receiver mounted on the utilized UAV platform. The image positions that are derived from the implemented SfM approach and the absolute orientation process is performed for the estimation of the 3D Helmert transformation parameters (i.e., scale factor, three translation parameters, and three rotation angles) relating the two involved coordinate systems. Although the imagery blocks of both epochs are now registered to the mapping frame coordinate system, it still does not satisfy the required level of accuracy required in this research. Therefore, a global bundle adjustment is performed using manually measured GCPs with ±3 cm accuracy. It should be noted that the GCPs are not required for all epochs, since the imagery from all the epochs are integrated in one block bundle adjustment.
4. The fourth step is semi-global dense matching procedure implemented to generate a dense 3D point cloud for each observed epoch using images captured only at that particular epoch. Note that the separate point clouds are now effectively co-registered to a common reference frame as a result of the bundle adjustment procedure.

### 3.3. Evaluation of The Proposed Registration Method

**Figure 4:**
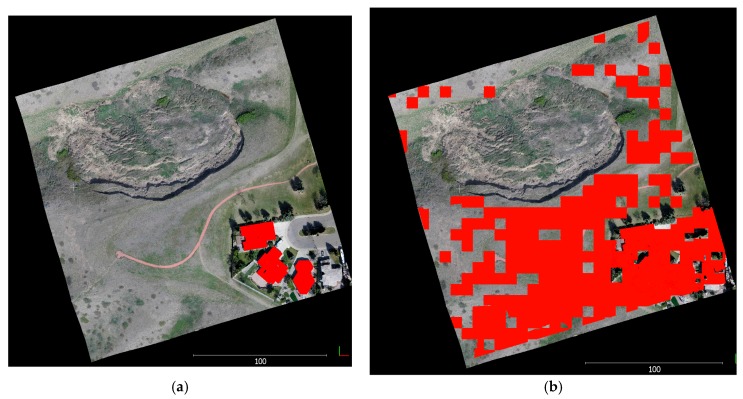
Selected patches used to estimate quantitative quality control overlaid on the point cloud. (**a**) Selected building rooftops; (**b**) selected stable area surfaces.

In this section, the qualitative and quantitative aspects of the proposed registration method versus the ICPP method are evaluated. Image-based point clouds are generated for each epoch separately (i.e., SfM and semi-global matching (SGM) for each epoch were processed individually) to implement the ICPP registration approach. Then, both resulting point clouds were registered to a common coordinate system using ICPP. Qualitative control is achieved by plotting together all the registered 3D dense image-based point clouds to the reference coordinate system. By examining the registered datasets more closely, the quality of the proposed registration method is evaluated, and a more detailed analysis is conducted using overlapping, stable, non-active areas between the 3D dense image-based point cloud datasets. Quantitative quality control of the registration method is necessary because the overlap area is affected by a landslide in this dissertation. In this case, the accuracy of the co-registered surfaces is estimated by comparing the non-active patches within the monitored area of interest. Since these non-active sub-areas are stationary, the two surfaces generated from different epochs theoretically should be close to each other. The quantitative quality control process is based on point-to-plane normal distances. These distances are calculated between registered point clouds generated using the proposed registration method and ICPP. The calculation steps are as follows:

1. The conjugate features in the non-active areas, including building rooftops and stable bare earth surfaces, are extracted. Building rooftops were extracted manually from both point clouds (see Figure 4a) and stable bare earth surfaces are extracted automatically based on the iterative closest proximity algorithm (ICProx-algorithm) proposed by Wujanz [31] (see Figure 4b). The ICProx-algorithm follows a geometrically-motivated strategy to overcome the low breakdown point of the L2-norm and to identify geometrically stable areas that are suitable for computation of the transformation parameters within point clouds. This is achieved by a bottom-up approach that divides the point clouds into spatially equal large segments by using an octree structure [38]. The basic idea is to locally increase the influence of outliers in order to identify them based on suitable criteria.
  All the segments are individually aligned via ICP, which leads to different sets of transformation parameters. Based on this outcome, congruent areas are identified by suitable criteria (e.g., A point from scan 1 needs to be located within the area that is described by its corresponding point and two adjacent points; a corresponding point has to lie on the surface that has been described in the previous step; and, at last, a corresponding point needs to be located within a partial area that has been derived from the area described by the first criterion). Finally, transformation parameters are determined by using the largest congruent cluster, which then is used integrally rather than in segments as during the diagnostic phase. This assumption can be made as the arbitrary deformation of an object or area leads to inconsistent changes and consequently different transformation parameters.
2. The root mean square error (RMSE), mean, and standard deviation of the calculated point-to-plane normal distances for each plane are calculated using Equations (1)–(3), respectively.

(1) $$RMSE=\sqrt{\frac{\sum {\left(nd\right)}^{2}}{n}}$$

(2) $$Mean=\bar{nd}=\frac{\sum nd}{n}$$

(3) $$Standard\;Deviation=\sigma =\sqrt{\frac{\sum {\left(nd-\bar{nd}\right)}^{2}}{n}}$$

where (*nd*) refers to the calculated normal distance between the points within the surface reconstructed from images captured in May 2014 and the surface from images captured in May 2015; (*n*) is the number of points within a selected surface.

### 3.4. Volumetric Change Detection Analysis

The high resolution of the UAV image-based point cloud (less than 10 cm/pixel) is the most significant advantage of the proposed method and the level of detail it provides allows for the investigation of changes that occur on the surface of the landslide-prone areas. 3D temporal variations of the terrain surface can be detected by comparing sequential point clouds obtained from UAV image data sets using SGM techniques. Exact point-to-point correspondence usually cannot be assumed because of the irregular nature of the point clouds. Therefore, geometric primitives were chosen using points and closest triangular patches [23]. The algorithm is based on the ICPatch/point-to-surface-based method, establishing correspondence between two surfaces by identifying conjugate surface features and then estimating the normal distances between these features. The proposed method uses the full density of the point cloud as only one of the point clouds requires a surface representation, thus saves computation time when compared to the point-to-point method [39]. Furthermore, change detection is calculated for every point in the point cloud.

This process was comprised of three steps: (1) reconstruction of the triangulated irregular network (TIN) surface of the first point cloud; (2) estimation of the normal distances between each point in the second point cloud and the closest patch in the first point cloud; and (3) evaluating the normal distance for each period against a predefined threshold to classify the points from the temporal epochs as match or non-match. Then, the percentage of non-match points was used to decide whether the surface moved according to a predefined threshold, which depended on the size and nature of the site. The amount of movement was computed as the average normal distance between non-match points.

### 3.5. Change Detection Analysis in The Horizontal Direction

**Figure 5:**
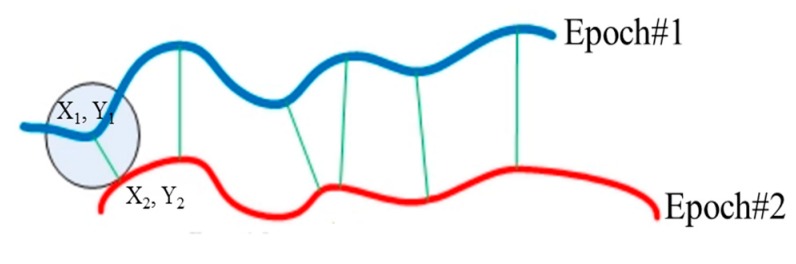
The procedure of calculating the horizontal displacement of the scarp retreat between the extracted epoch’s landslide scarps.

Horizontal displacement or scarp retreat was estimated using the extracted landslide scarps in each epoch based on the ratio of the normalized eigenvalues (i.e., $\lambda_{1}/\lambda_{2}\geq \lambda_{3}$) derived using principal component analysis (PCA) from a 3D image-based point cloud as explained by Al-Rawabdeh et al., 2016 [40] (see Supplementary Materials for a brief summary of methodology used in ref [40]). Figure 5 indicates the landslide scarp retreat calculated by estimating the Euclidian distance between each point in one of the extracted epoch’s landslide scarp and the correspondence closest point in the second scarp (second epoch). The RMSE of the estimated distance was then calculated to represent the horizontal displacement of the scarp retreat between the epoch’s landslide scarps.

## 4. Results and Discussion

The registration results were evaluated qualitatively as well as quantitatively. An evaluation of the change detection method in two directions is described in this section using the original image-based point clouds.

### 4.1. Data Description

**Figure 6:**
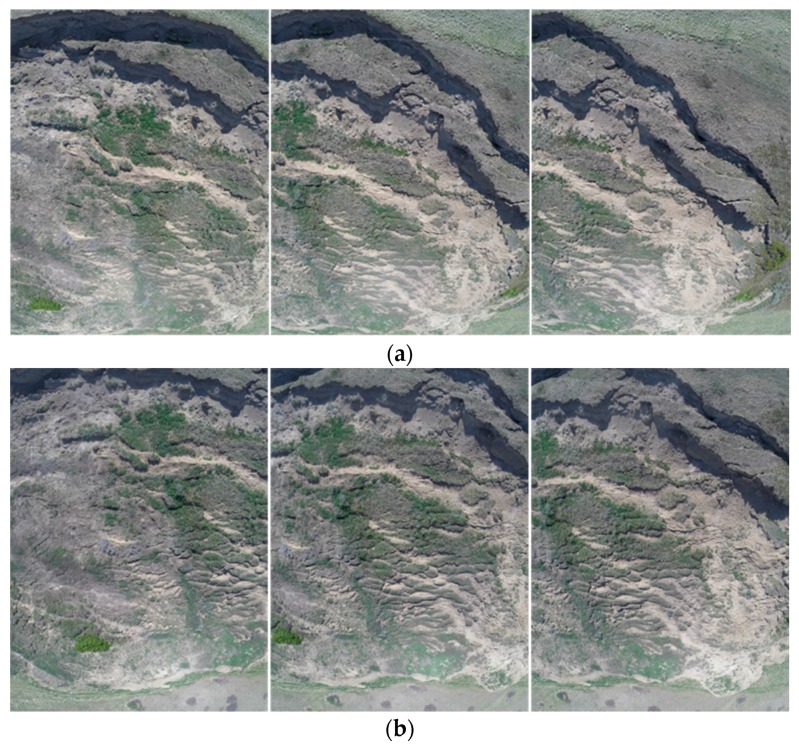
Sample set of UAV images of the experimental dataset: (**a**,**b**) showing sequence images with 80% overlap along the flight path, as well as showing the sidelap between adjacent flight paths.

The autonomous flight was conducted at an altitude of roughly 30 m above ground level (AGL) and at a speed of 5 m/s for each of the four flight missions (Figure 6 and Table 1) during two separate expeditions to the Lethbridge site. The study area was covered following a grid of parallel and perpendicular flight lines (north–south and east–west) ensuring that each ground object was imaged in the along and across-track directions of the UAV platform for maximizing overlap. The flights over the study area covered an area of approximately 0.04 km^2^. The GSD achieved was approximately 1.7 cm at an average altitude of 27 m AGL.

### 4.2. 3D Surface Reconstruction

**Figure 7:**
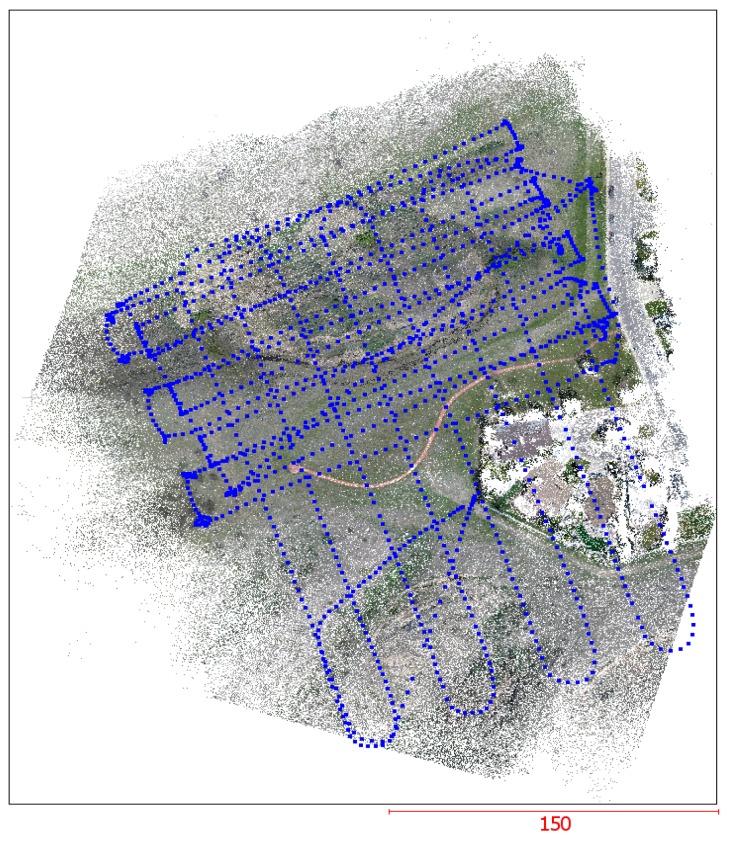
A perspective view of the constructed sparse point cloud. The blue dots over the sparse points show the camera’s position during the image acquisition by the UAV.

The proposed procedure using the adopted SfM for the automated EOPs recovery was tested using both image datasets from both time periods within the global block bundle adjustment as one processing unit. Within the SfM procedure, 712 images from the original set of 997 images taken in May 2014 and 831 images from the 1241 images taken in May 2015 for the Lethbridge study area were selected after the blurred and highly-overlapped images were removed to reduce the computational cost. An example of the UAV image dataset, estimated image positions, and orientations, as well as the reconstructed sparse point cloud of all the remaining images with respect to the mapping frame coordinate system are shown in Figure 7. As summarized in Table 2, the image re-projection errors tend to be approximately one pixel for both image datasets. These results indicate that the EOPs estimated through the proposed procedure are accurate. Moreover, the results also indicate that the proposed procedure also can handle sets of collected images from different times.

**Figure 8:**
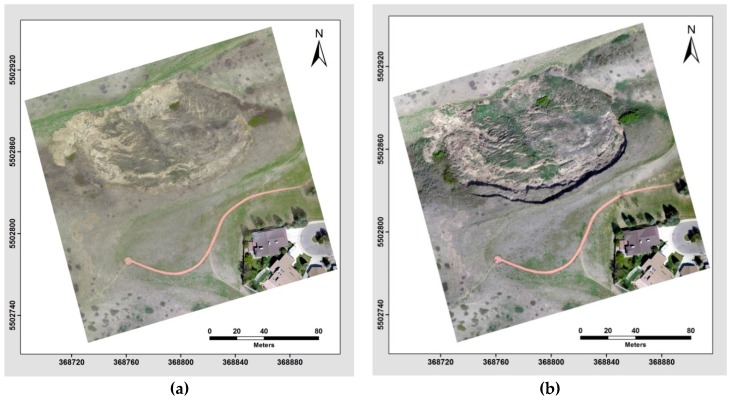
Perspective view of the colourized dense 3D UAV image-based point cloud generated using the semi-global matching (SGM) algorithm, the point cloud from the image sets captured on May 2014 (**b**) and the point cloud from the image sets captured on May 2015 (**a**).

The point clouds generated from selected images of the study site from May 2014 and May 2015 were comprised of more than 18 million and 19 million points, respectively. The density of these point clouds was approximately 4000 points per square meter (m^2^), and the resulting point clouds from the different epochs are shown in Figure 8.

### 4.3. Quality Control of the Registration Results

**Figure 9:**
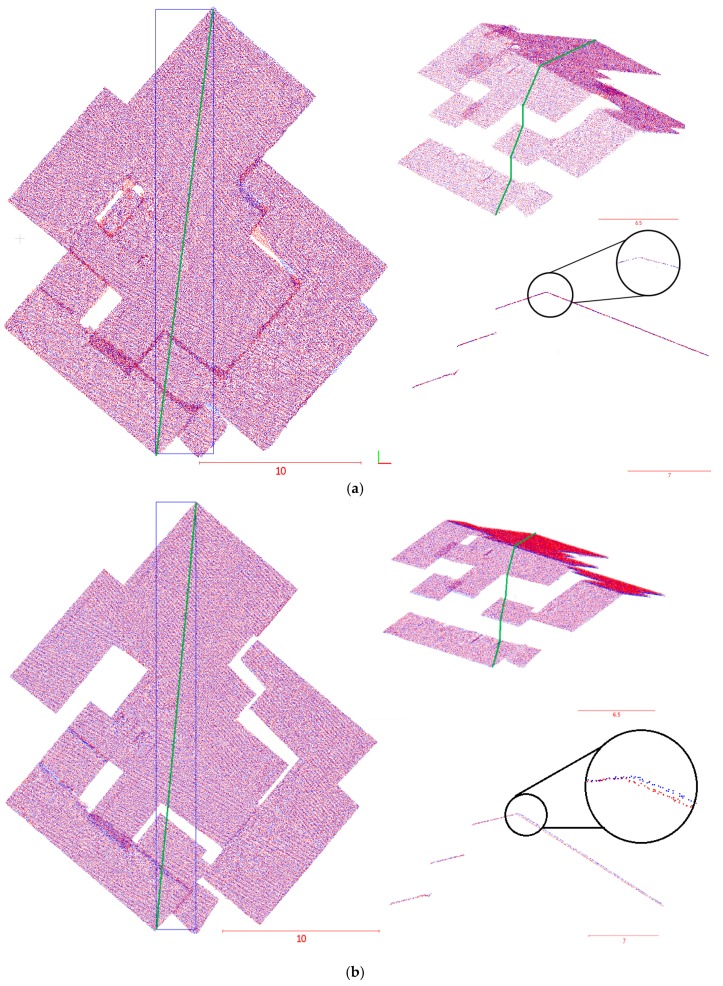
Registration results for a part of the Lethbridge landslide area. (**a**) Results based on the proposed registration method; (**b**) results based on the iterative closest projected point (ICPP)-based registration method.

The registration results were evaluated for qualitative control, whereby the 3D dense image-based point cloud surfaces were registered and were plotted together. To obtain a more detailed analysis of the results, closer examination of the registered dense point clouds was required. Given that most of the overlapping areas between the two datasets were affected by the landslide, only a few overlapping parts of the two registered datasets were closely evaluated. The common features present in the overlap areas, such as the building’s rooftops in the registered datasets, are illustrated in Figure 9. In this figure, each colour indicates a different 3D image-based point cloud generated at a different time. Figure 8a,b display the registration results of selected parts of the rooftops based on the proposed registration method and the ICPP-based registration method, respectively. It is clearly visible in Figure 8b that the registration between the point clouds from two epochs, based on the ICPP method, did not align the clouds correctly.

**Figure 10:**
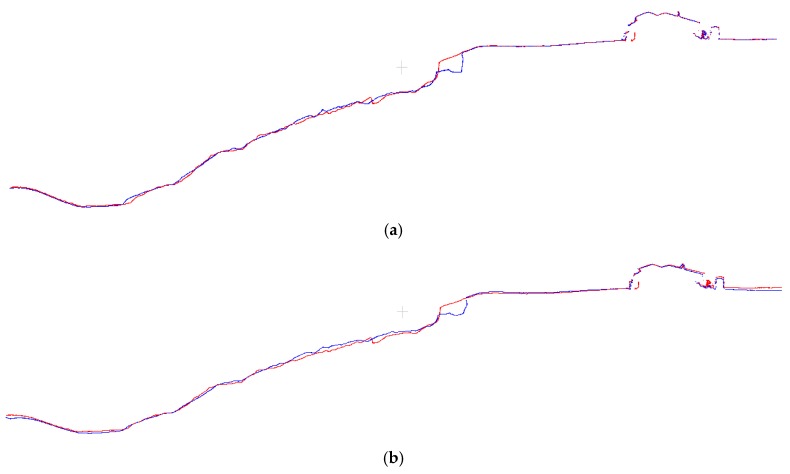
Registration results of a cross-section diagonally across the Lethbridge landslide area between the epoch from 2014 (red) and the epoch captured in 2015 (blue). (**a**) Cross-section along the study area after the registration using the proposed registration method; (**b**) Cross-section along the study area after the registration using ICPP-based registration method.

Cross-sectional views of the same sections between the generated point clouds are shown in Figure 10a,b, and provide a better visualization of the registration results. As illustrated in Figure 9a, the 3D dense image-based point clouds are appropriately registered using the proposed method. In contrast, Figure 9b demonstrates that the ICPP registration method was less successful than the proposed method in establishing a precise alignment between the two point clouds.

The quantitative analysis of the registration results was achieved by calculating the point to plane normal distances for the selected planes between two generated 3D image-based point cloud surfaces over one year. Using the proposed registration method, all the dense point cloud surfaces were registered with respect to a common coordinate system. The calculated mean, standard deviation, and RMSE of the point to-plane normal distances between the two sets of point clouds are presented in Table 3.

**Figure 11:**
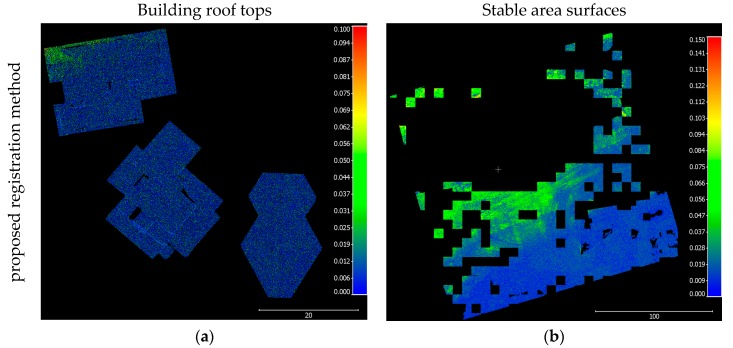

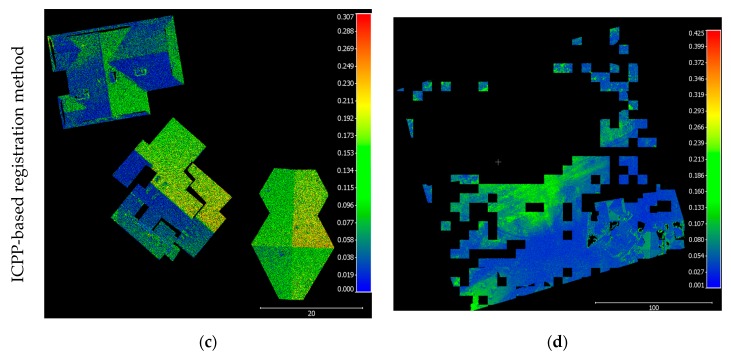
The top views of the two registered 3D dense image-based point clouds over the span of one year utilizing selected patches from the Lethbridge, Alberta, datasets to estimate quantitative quality control. The colours represent the normal distances calculated using the different methods. (**a**) Proposed registration method using the building roof tops point cloud; (**b**) Proposed registration method using the stable area surfaces point cloud; (**c**) ICPP-based registration method using the building roof tops point cloud; and (**d**) ICPP-based registration method using the stable area surfaces point cloud.

Analysis of the normal distance results for each of the selected patches indicated that the proposed registration method can achieve an accurate alignment between the multi-temporal point clouds. The mean, standard deviation, and RMSE of the calculated point to-plane normal distances of each sample patches were all below 4 cm approximately, substantiating the quality of the registration results in Table 3. However, it was noted that the results of the calculated normal distances using the points of the building rooftops located in the region of the stable area and was unaffected by vegetation growth were generally smaller than the calculated normal distances using all the points of the selected stable bare ground area. Finally, a visual illustration of the calculated point-to-plane normal distances was completed by plotting the point clouds over one year using colours based on the normal distance in Figure 11. From these results, it was concluded that the datasets for the proposed method were registered accurately.

### 4.4. Change Detection Analysis

**Figure 12:**
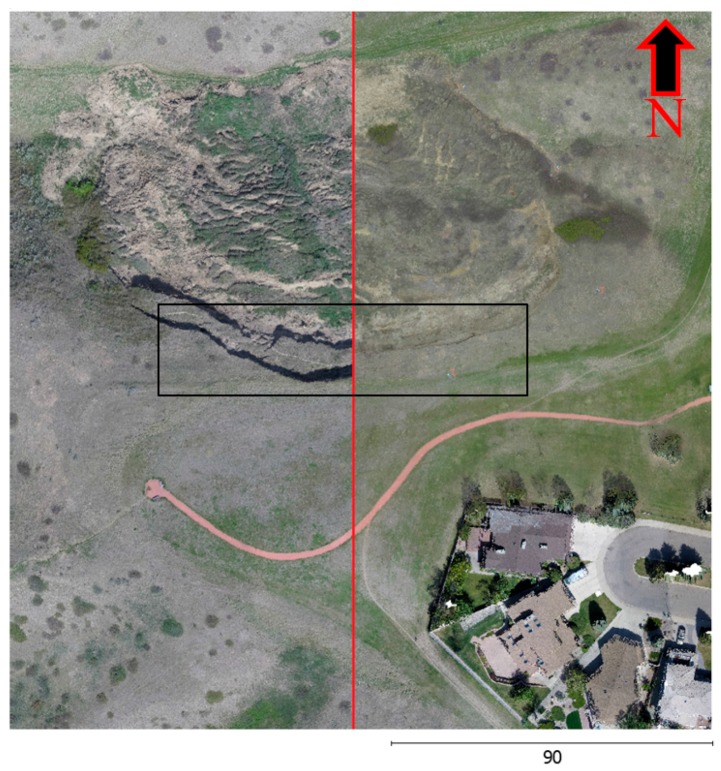
Visual comparison of the two periods, (Left, 2015) and (Right, 2014).

The results of the change detection analysis procedure, in both the non-horizontal and horizontal directions, on the landslide area located in southern Lethbridge, Alberta, are presented in this section. The dynamics of the landslide between two epochs were visually investigated by overlaying two colored 3D image-based point clouds to easily recognize any displacements on the screen. The results were visible in the colorized point clouds after the registration in Figure 12. This illustration is divided into two parts by a red line: the left part shows data from 2015, and the right part depicts data from 2014. The breaking edge’s expansion by about 5.5 m towards the residential area is clearly evident at the center of the picture within the black rectangle.

For the results presented in Figure 12, a visual observation using Google Maps indicated that the landslide developed in 2000 and continues to evolve. Field and aerial surveys revealed that the main scarp developed approximately 4 m backwards and the northern toe extended by approximately 3.5 m between May 2014, and May 2015.

Point-to-patch comparison allowed volumetric surfaces changes to be analyzed. The extracted landslide scarps from each generated point cloud were used to estimate the horizontal rate displacements. The horizontal displacement rate results are described in detail in the following sub-sections.

#### 4.4.1. Volumetric Change Detection Measurements Analysis

**Figure 13:**
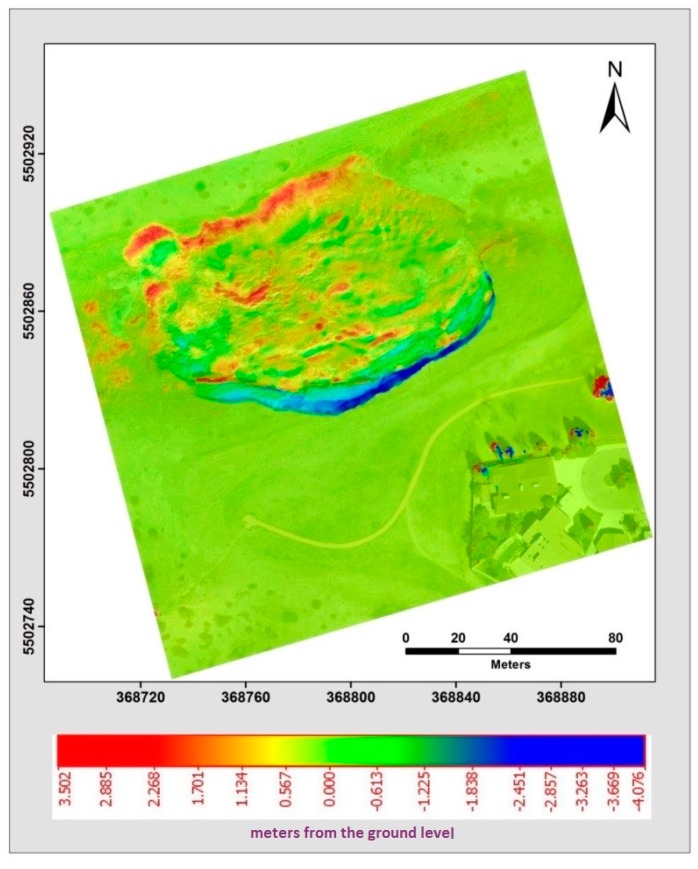
Illustrates the height difference in meters between the point cloud surfaces created from low altitude UAV imagery obtained in two different epochs in May 2014 and May 2015. For each point, the distance, from the ground level on top of the landslide is either positive (red) due to deposition or negative (blue) due to erosion.

The results from the volumetric change detection between the point clouds created from two different epochs are demonstrated in Figure 13. A point cloud generated from UAV imagery over the study area from 2014 was used as the triangular irregular network (TIN) surface and was compared with the corresponding dataset from 2015 using the point-to-patch comparison. The changes between these two datasets were detected on an individual point basis by computing the normal distances for all the points in one dataset to their respective nearest patch from the other. During the change detection process, a significant level of volumetric displacement variation between datasets was evident. Changes within the area were partially caused by a section of the landslide scarp was eroded and was placed at the bottom, or toe, of the landslide which is called deposition. These changes can be seen in Figure 13, where the dark blue area represents a volumetric terrain loss and the red area represents an increase in the volumetric terrain. The points are considered to be a possible detected change if the normal distance exceeded a predefined threshold; in this case, it was established as 15 cm based on the characterization of study area. Areas of minor change can be seen in Figure 13. Review of these areas against the point cloud datasets revealed that these changes were false identifications, related to errors due to vegetation growth/trees.

#### 4.4.2. Displacements Measurements Analysis in Horizontal Direction

After precise co-registration, based on the proposed practical registration method of the point clouds, the horizontal surface displacement of the landslide was computed as a Euclidian distance between the extracted successive landslide scarps over time. The extracted scarp segments were derived using advanced methods based on geomorphological factors developed by Al-Rawabdeh et al. [40]. These methods are referred to as the ratio of the normalized eigenvalues (i.e., $\lambda_{1}/\lambda_{2}\geq \lambda_{3}$) derived using principal component analysis (PCA) from a 3D image-based point cloud (Supplementary Materials).

The following results demonstrate the process for detecting and extracting main landslide scarps from a single surface using an image-based point cloud generated using the SGM approach. The extracted successive landslide scarps between the two datasets were used to determine horizontal displacement over time.

**Figure 14:**
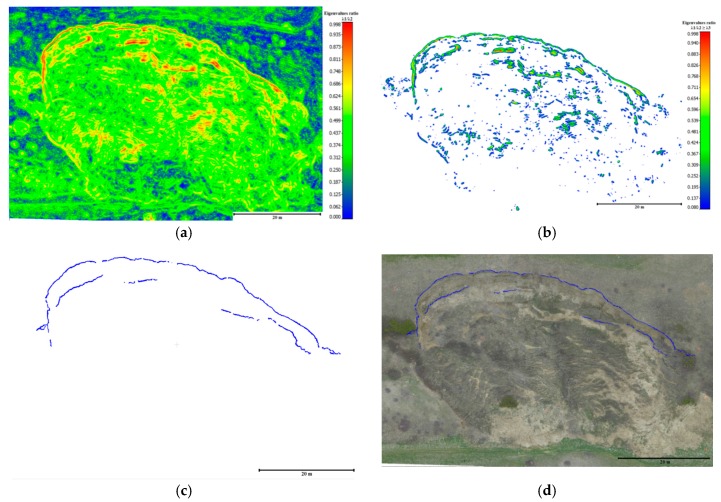
Extracted landslide scarps using eigenvalue ratio ($\lambda_{1}/\lambda_{2}\geq \lambda_{3}$) computed using the dense 3D image-based point cloud dataset collected in May 2014 for the study landslide, Lethbridge, Alberta. (**a**) Eigenvalue ratio (λ1/λ2); (**b**) extraction of landslide scarp features based on variation in the local topography’s eigenvalue ratio; (**c**) final landslide scarp detection results based on eigenvalues ratios of the topographic surface index; (**d**) scarps overlaid on the orthophoto using UAV images of the study area.

**Figure 15:**
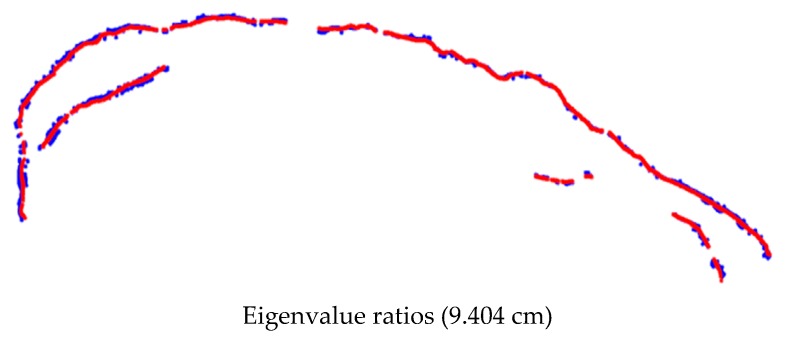
The discrepancies between the ground truth (red) and the extracted landslide scarp data collected using the data collected in May 2014 (blue).

**Figure 16:**
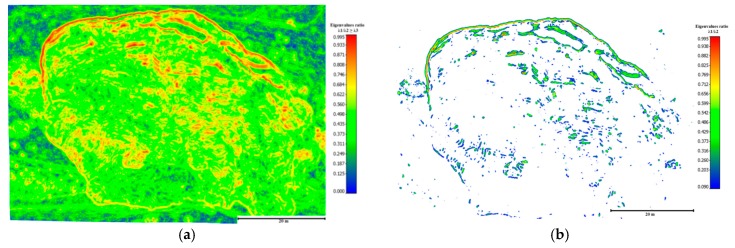

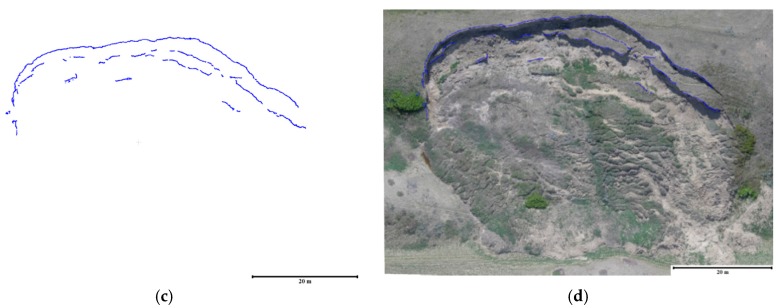
Extracted landslide scarps using eigenvalue ratio ($\lambda_{1}/\lambda_{2}\geq \lambda_{3}$) computed using the dense 3D image-based point cloud dataset collected in May 2015 for the study landslide, Lethbridge, Alberta. (**a**) Eigenvalue ratio (λ1/λ2); (**b**) extraction of landslide scarp features based on variation in the local topography’s eigenvalue ratio; (**c**) final landslide scarp detection results based on eigenvalues ratios of the topographic surface index; (**d**) scarps overlaid on the orthophoto using UAV images of the study area.

**Figure 17:**
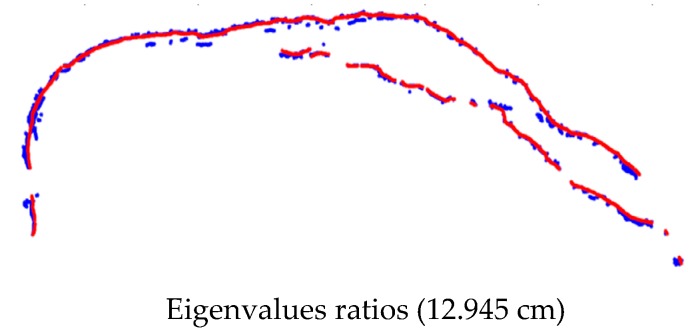
The discrepancies between the ground truth (red) and the extracted landslide scarp data collected using the data collected in May 2015 (blue).

Figure 14 depicts an example of the landside’s main scarp extraction performed by the normalized Eigenvalue ratio using the point clouds from images acquired in May 2014. Figure 15 indicates the discrepancies between the ground truth and the extracted landslide scarp. The same procedure was also implemented for extracting the landslide main-scarp using the UAV image-based point clouds from images gathered in the May 2015 effort (see Figure 16 and Figure 17).

The overall quality of the landslide scarp segments extracted from the site over the two datasets were calculated based on the confusion matrices and their RMSE, which are summarized in Table 4 and Table 5, respectively.

**Figure 18:**
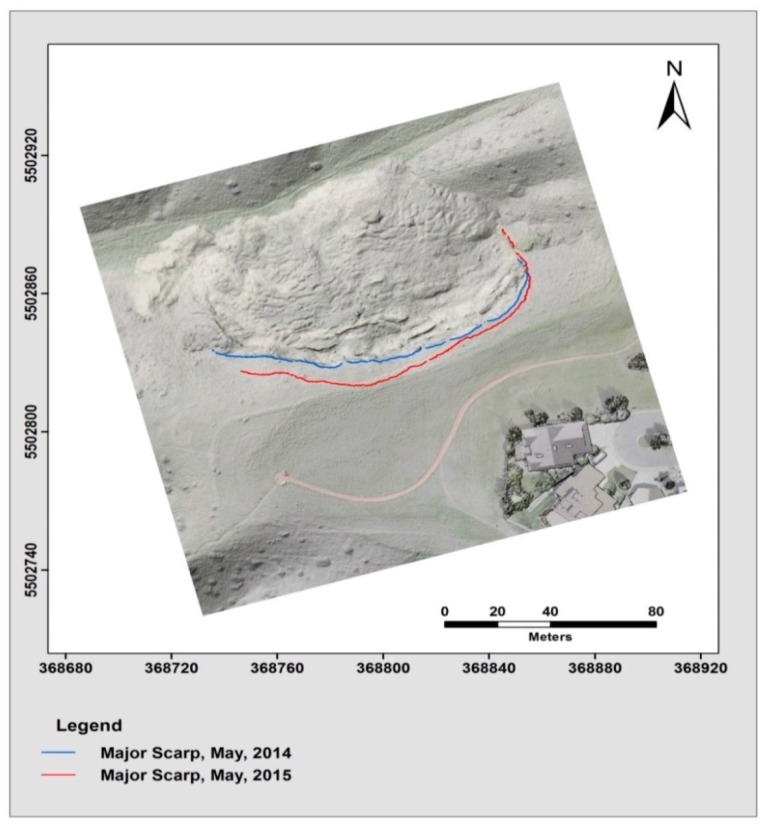
Illustrates the extracted landslide scarp feature results for an epoch from 2014 (blue) and an epoch captured in 2015 (red) of the Lethbridge, Kings Park landslide area.

**Figure 19:**
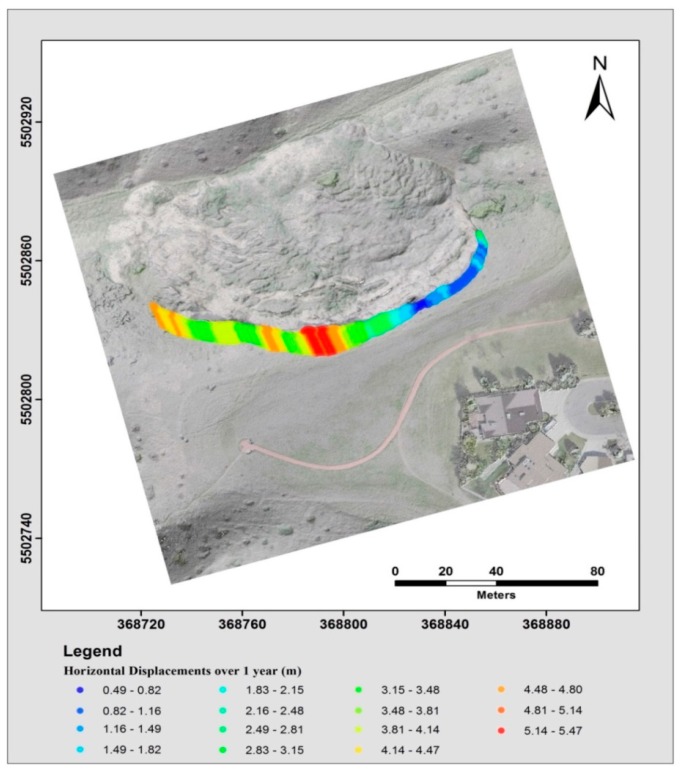
Horizontal displacement difference, scarp retreat, between the extracted scarps of May 2014 and May 2015 illustrating the surface changes and, therefore, the dynamics of the landslide.

The results of the visual interpretation of the landslide displacement based on the comparison of the extracted landslide main-scarps from different epochs were overlaid on the orthomosaic of May 2014, which is presented in Figure 18. The red color represents the main-scarp extracted from the point cloud on May 2015, and the blue color shows the scarps extracted from the May 2014 point cloud. The computed displacement was visualized using a multiple colour-coded illustration highlighting the magnitude of the displacement from the comparison between the extracted scarps of May 2014 and May 2015 (see Figure 19). The significant retreat of the main scarp indicates the displacement of the landslide material was not equally distributed over the slide and differed significantly from one location to another. The eastern part of the main scarp retreated by approximately 1 m, whereas the middle section was the most active and expanded more than 5 m.

## 5. Conclusions

Utilizing point clouds that were derived from imagery captured by a low-cost UAV, this research presented a practical approach for the detection of landslides. Utilizing the proposed methodology was effective in acquiring and deriving accurate information for landslide characterization, reducing surveying hazards during the data collection stage, and reducing overall incurred costs. Eigenvalue ratios in the local neighborhood were used in the automated approach with a 0.5 m search radius to extract the salient geo-morphometric features that represented the scarps which were utilized. Results show that the proposed approach can accurately identify and extract landslide scarps with a recognition accuracy based on Cohen’s kappa coefficient, which, using the data from 2014, was 81%, scarp detection was 94% complete, 74.6% correct, and of 96% overall quality. Recognition accuracy based on Cohen’s kappa coefficient using the data from 2015 was 71%, scarp detection was 96.6% complete, 69% correct, and of 95.7% overall quality. These measurements were evaluated by calculating the RMSE between the automatically-extracted landslide scarp segments and the manually-digitized ones. The RMSE analyses also revealed that the accuracy of the Eigenvalue-based approaches was 9.4 cm in 2014 and 12.9 cm in 2015.

The ability to detect landslide scarps will lead to a better understanding of landslide mechanisms for a given area, thus leading to an enhanced identification of the most likely failure sites within a landslide-prone area. It also can assist in estimating the volume of the potential sliding of the Earth’s surfaces. In this study, a novel practical approach was introduced for detecting change with a high degree of precision in multi-temporal UAV image-based point clouds. The dense 3D point cloud datasets were generated using the acquired temporal series images obtained in a flight mission covering an active soil creep landslide area in Lethbridge, Alberta, in two epochs over a period of one year (May 2014 and May 2015). The developed registration method is robust and automated, which is paramount for evaluating the effectiveness of change detection to identify and quantify movement in areas of erosion or deposition caused by rain events, as well as allowing for regular updating of topographic data in landslide areas prone to rapid change. The proposed approach can easily process large numbers of images from different epochs and enables the provision of registered image-based point clouds without the use of extensive ground control point information. The accuracy of co-registered surfaces was validated by comparing non-active patches within the monitored area of interest. Since these non-active sub-areas were stationary, the computed normal distances should theoretically be close to zero. The quality control of the registration results showed that the average normal distance was approximately 3.7 cm, which is within the noise level of the reconstructed surfaces. The resulting change detection information is sufficient and could be used by municipal authorities to contribute to their urban planning and decision making. A disadvantage and extension for future work will be to address areas covered by bushes, boulders, and tree stumps, and more specifically, developing filtering techniques to remove such features before the application of the proposed approaches.

Overall, it can be concluded that the developed registration approach is low level, i.e., the registration is performed at the image/bundle adjustment level as opposed to the point cloud level. Compared with the ICPP registration method, the developed method in this research takes full advantage of automated geometric measuring, leading to fast processing. It is also economical, labor-saving, and includes safe tools for detecting and recognizing landslide scarps. The produced results are suitable for use in the field of landslide research to monitor and to assess the rate of horizontal displacement (horizontal scarp progression or scarp retreated) between the extracted landslide scarps at different times.

## Figures and Tables

**Table 1:** Overview of all performed UAV flights for the study area, Lethbridge, Alberta in May 2014 and May 2015.

Flight Date	Area Covered (km^2^)	Flight Direction	Duration (min)	Number of Images Taken	Number of Images Used
May 2014	0.0317	N–S	11.23	497	387
	0.0387	E–W	8.57	500	325
May 2015	0.0307	N–S	12.08	602	411
	0.0387	E–W	9.35	639	420

**Table 2:** Structure from motion (SfM) results using all the captured images over a period of one year.

No. of Images	1543	No. of Tie Points	1,054,494
Average Flying altitude	27 m	Image space error	1.33 pix
Ground resolution	0.02 m/pix	Coverage area	0.044 km^2^

**Table 3:** The mean, standard deviation, and RMSE of the calculated normal distances results of the proposed registration method and the ICPP for the stable patches between 3D dense image-based point clouds generated in May 2014 and May 2015 of Lethbridge, Alberta study area.

Registration Methods		Building Rooftops	Stable Area Surfaces
Proposed Method	Mean (mm)	0.8	24.9
	Standard deviation (mm)	15.2	27.3
	RMSE (mm)	15.3	36.9
ICPP Method	Mean (mm)	55.9	60.7
	Standard deviation (mm)	46.2	32.4
	RMSE (mm)	67.5	68.8

**Table 4:** Comparison of the statistical quality assessment results for landslide scarp segments extracted from the Kings Park, Lethbridge landslide study area from both datasets, May 2014 and May 2015.

Dataset	Algorithm Approache	Overall Quality (%)	Completeness (%) (Producer’s Accuracy)	Correctness (%) (User’s Accuracy)	Kappa Coefficient (%)	Degree of Agreement
May 2014	Topographic surface Eigenvalue ratios	96.1	94.03	74.62	81	Very good
May 2015		95.74	96.6	69.10	71.31	Very good

**Table 5:** The RMSE between the ground truth and the extracted landslide scarp using the different approaches for datasets of the study landslide, May 2014 and May 2015.

Dataset	Algorithms Approaches	RMSE (cm)
May 2014	Topographic surface Eigenvalue ratios	9.404
May 2015	Topographic surface Eigenvalue ratios	12.945

## Supplementary Materials

The following are available online at www.mdpi.com/1424-8220/17/10/2378/s1.
