# Supplementary material for: Time Series UAV Image-Based Point Clouds for Landslide Progression Evaluation Applications
Source: Sensors (Basel). 2017 Oct 18;17(10):2378. doi: 10.3390/s17102378 (PMC5677266; doi:10.3390/s17102378)
Supplement: Supplementary file 1 [file sensors-17-02378-s001.pdf]

APPENDIX

Eigenvalue Ratio Analysis:

Eigenvalue ratios represent the degree of three-dimensional roughness or the crease edge of land surfaces [1]. Point clouds that result from the SGM approach are comprised of massive amounts of 3D coordinates. The KD-tree data structure [2] was used to handle the point cloud, which is a tool for organizing the point cloud and allows for different query processes in the 3D space during the Eigenvalue estimating procedure. The KD-tree is used to search for neighbors within a specified search radius from the query point. A simple method for establishing this local neighborhood is to select the closest points to the query point according to a fixed Euclidian distance. 0.5 m radius was selected in this study because of the size of the relevant geomorphic features. Smaller radius would have resulted in an increase in recognition of non-scarp landscape features, such as shrub vegetation, tree stumps or boulders. The Eigenvalue ratio methodology steps are i) utilizing PCA to determine the geometric properties of the local neighborhood of image-based points (To check whether a certain point (query point) belongs to a rough surface or a crease edge); ii) defining a local neighborhood (P_n) to enclose the (n) neighbors nearest to the query point; iii) a covariance matrix (Cov) is formed based on the dispersion of the points (P_n) from their centroid ($\bar{P}_{cX}$), as given by Equation (Equation 1). iv) Performing an eigenvalue analysis to decompose the covariance matrix into two matrices (Equation 2). The first matrix (W) is comprised of three eigenvectors ($\vec{e}_1, \vec{e}_2, \vec{e}_3$), and the other matrix (Λ) provides their corresponding Eigenvalues (λ1,λ2,λ3).

The Eigenvectors/Eigenvalues are quite helpful in determining the geometric nature of the established neighborhood. The Eigenvectors represent the orientation of the neighborhood in 3D space, while the Eigenvalues define the extent of the neighborhood along the directions of their corresponding eigenvectors [3]. The relative sizes of the Eigenvalues and the Eigenvectors' directions indicate the type of primitive feature. For a rough surface/crease edge point, two of the estimated Eigenvalues will be much smaller than to the third Eigenvalue, for which the conventional equations (Equations 3a and 3b) were used in this study. The three normalized Eigenvalues denoted by "λ" were sorted from largest to smallest values as λ_3, λ_2, and λ_1.

$$Cov_{3x3} = \frac{1}{n}\sum_{i=1}^{n}\left(\begin{bmatrix}P_{iX}\\P_{iY}\\P_{iZ}\end{bmatrix} - \begin{bmatrix}\bar{P}_{cX}\\\bar{P}_{cY}\\\bar{P}_{cZ}\end{bmatrix}\right) - \left(\begin{bmatrix}P_{iX}\\P_{iY}\\P_{iZ}\end{bmatrix} - \begin{bmatrix}\bar{P}_{cX}\\\bar{P}_{cY}\\\bar{P}_{cZ}\end{bmatrix}\right)^T \tag{1}$$

Where

$$P_c = \frac{1}{n}\sum_{i=1}^{n}\begin{bmatrix}P_{iX}\\P_{iY}\\P_{iZ}\end{bmatrix}$$

$$Cov_{3x3} = W\Lambda\,W^T = [\vec{e}_1\vec{e}_2\vec{e}_3]\begin{bmatrix}\lambda_1 & 0 & 0\\0 & \lambda_2 & 0\\0 & 0 & \lambda_3\end{bmatrix}\begin{bmatrix}\vec{e}_1^{\,T}\\\vec{e}_2^{\,T}\\\vec{e}_3^{\,T}\end{bmatrix} \tag{2}$$

$$\lambda_1 \approx \lambda_2 \tag{3a}$$

$$\frac{\lambda_1}{\lambda_2} \geq \lambda_3 \tag{3b}$$

Finally, the topographic parameters using the normalized Eigenvalue ratio of $\lambda1/\lambda2$ computed in a 0.5 m moving sampling window on a dense 3D image-based point cloud.

Quality Assessment Factors (Table 4):

The overall accuracy of the confusion matrix: dividing the total number of agreements (i.e., the sum of the diagonal cells of the matrix) by the total number of samples. The user's accuracy represents a measure of correctness (Equation 3), and the producer's accuracy represents a measure of completeness (Equation 4) [4-5].

$$Correctness = \left(\frac{TP}{TP+FP}\right) \times 100 \quad (3)$$

$$Completness = \left(\frac{TP}{TP+FN}\right) \times 100 \quad (4)$$

FP=false positive

FN= false negative

TP= true positive

Cohen's kappa coefficient was calculated from the confusion matrix, which estimated the performance evaluation of the landslide extraction. This coefficient was a measure of the agreement between the extracted and the referenced data. In other words, the kappa statistics were the measure of true agreement, which was represented by the following relationship [6].

$$Kappa\ Coefficient(k) = \frac{N\sum_{i=1}^{r} X_{ii} - \sum_{i=1}^{r}(X_{i+} * X_{+i})}{N^2 - \sum_{i=1}^{r}(X_{i+} * X_{+i})} \quad (5)$$

r = the number of rows in the confusion (error) matrix

X_{ii} = the number of observations in row *i* and column*i* on the major diagonal of the matrix

X_{i+}= the total observations in the row *i*

X_{+i}= the total observations in the column *i*

N = the total number of observations that were included in the matrix

Acronym Table

Acronym	Definition
AGL	Above Ground Level
COSI-Corr	Co-registration of Optically Sensed Images and Correlation
DEMs	Digital Elevation Models
DG	Direct Geo-Referencing
EOPs	Exterior Orientation Parameters
GCPs	Ground Control Points
GNSS	Global Navigation Satellite System
GPS	Global Positioning System

GSD	Ground Sample Distance
ICP	Iterative Closest Point
ICPP	Iterative Closest Projected Point
INS	Internal Navigation System
IOPs	Interior Orientation Parameters
LFOV	Low-Cost Large-Field-Of-View
LiDAR	Light Detection And Ranging
MVS	Multi-View Stereopsis
PCA	Principal Component Analysis
PMVS	Patch-Based Multiview Stereo
ROPs	The Relative Orientation Parameters
SfM	Structure from Motion
SGM	Semi-Global Matching
SIFT	Scale-Invariant Feature Transform
SMAC	Simultaneous Multi-Frame Analytical Calibration
RMSE	Root-Mean-Square Error
TIN	Triangular Irregular Network
TLS	Terrestrial Laser Scanning
UAVs	Unmanned Aerial Vehicles
USGS	United States Geological Society

REFERENCES

1- Kasai, M.; Ikeda, M.; Asahina, T.; Fujisawa, K. (2009). LiDAR-derived DEM evaluation of deep-seated landslides in a steep and rocky region of Japan. Geomorphology, 113, 57–69.

2- Bentley, J. (1975). Multidimensional binary search trees used for associative searching. Communications of the ACM, 18 (9), 509–517.

3- Pauly, M., Gross, M., &Kobbelt, L. P. (2002, October). Efficient simplification of point-sampled surfaces. In Proceedings of the conference on Visualization'02 (pp. 163-170). IEEE Computer Society.

4- Zhan, Q., Molenaar, M., Tempfli, K., Shi, W., Shi, W., &Tempfli, K. (2005). Quality assessment for geo-spatial objects derived from remotely sensed data. International Journal of Remote Sensing, 26 (14), 2953–2974.

5- Tuermer, S., Kurz, F., Reinartz, P., &Stilla, U. (2013). Airborne vehicle detection in dense urban areas using HoG features and disparity maps. IEEE Journal of Selected Topics in Applied Earth Observations and Remote Sensing, 6(6), 2327-2337.

6- Sim, J., & Wright, C. (2005). The kappa statistic in reliability studies: use, interpretation, and sample size requirements. Physical Therapy, 85 (3), 257–268.
